# Induction of Long-term Depression-like Plasticity by Pairings of Motor Imagination and Peripheral Electrical Stimulation

**DOI:** 10.3389/fnhum.2015.00644

**Published:** 2015-12-01

**Authors:** Mads Jochumsen, Nada Signal, Rasmus W. Nedergaard, Denise Taylor, Heidi Haavik, Imran K. Niazi

**Affiliations:** ^1^Center for Sensory-Motor Interaction, Department of Health Science and Technology, Aalborg University DenmarkAalborg, Denmark; ^2^Health and Rehabilitation Research Institute, Auckland University of TechnologyAuckland, New Zealand; ^3^Center for Chiropractic Research, New Zealand College of ChiropracticAuckland, New Zealand

**Keywords:** long-term depression, long-term potentiation, cortical excitability, contingent negative variation, brain plasticity, associative stimulation, motor imagination

## Abstract

Long-term depression (LTD) and long-term potentiation (LTP)-like plasticity are models of synaptic plasticity which have been associated with memory and learning. The induction of LTD and LTP-like plasticity, using different stimulation protocols, has been proposed as a means of addressing abnormalities in cortical excitability associated with conditions such as focal hand dystonia and stroke. The aim of this study was to investigate whether the excitability of the cortical projections to the tibialis anterior (TA) muscle could be decreased when dorsiflexion of the ankle joint was imagined and paired with peripheral electrical stimulation (ES) of the nerve supplying the antagonist soleus muscle. The effect of stimulus timing was evaluated by comparing paired stimulation timed to reach the cortex before, at and after the onset of imagined movement. Fourteen healthy subjects participated in six experimental sessions held on non-consecutive days. The timing of stimulation delivery was determined oﬄine based on the contingent negative variation (CNV) of electroencephalography brain data obtained during imagined dorsiflexion. Afferent stimulation was provided via a single pulse ES to the peripheral nerve paired, based on the CNV, with motor imagination of ankle dorsiflexion. A significant decrease (*P* = 0.001) in the excitability of the cortical projection of TA was observed when the afferent volley from the ES of the tibial nerve (TN) reached the cortex at the onset of motor imagination based on the CNV. When TN stimulation was delivered before (*P* = 0.62), or after (*P* = 0.23) imagined movement onset there was no significant effect. Nor was a significant effect found when ES of the TN was applied independent of imagined movement (*P* = 0.45). Therefore, the excitability of the cortical projection to a muscle can be inhibited when ES of the nerve supplying the antagonist muscle is precisely paired with the onset of imagined movement.

## Introduction

Long-term potentiation (LTP) and long-term depression (LTD) are thought to be responsible for the synaptic changes associated with learning and memory (Malenka and Bear, 2004; Pascual Leone et al., 2005). However, damage to the central nervous system and deficient homeostatic mechanisms (possibly metaplasticity or synaptic scaling) cause changes, where the excitability of neural circuits may be abnormally increased or decreased (Quartarone et al., 2005; Weise et al., 2006; Kang et al., 2011). Therefore, artificial induction of LTP-like or LTD-like plasticity in the nervous system has been proposed as a means of addressing impairments such as paresis and spasticity after stroke and cerebral palsy (Dietz and Sinkjaer, 2007; Castel-Lacanal et al., 2009), as well as for conditions such as focal hand dystonia (Byl, 2007). Different methods for non-invasive stimulation of the brain have been proposed such as: anodal or cathodal transcranial direct current stimulation (Nitsche and Paulus, 2000), low- or high-frequency repetitive transcranial magnetic stimulation (TMS; Pascual-Leone et al., 1994; Chen et al., 1997), theta burst stimulation (Stefan et al., 2008) and paired associative stimulation (PAS; Stefan et al., 2000; Wolters et al., 2003; Chisari et al., 2014). Using these protocols bidirectional changes in cortical excitability have been demonstrated, where the excitability can be up or down regulated dependent on the stimulation method and parameters.

Traditional PAS protocols involve pairing of an afferent electrical stimulus with cortical activation induced by TMS. Recently, a novel PAS protocol was proposed, where the cortical activation induced by TMS is replaced by the naturally occurring cortical activation associated with motor imagery (Walter et al., 1964). During both cued motor imagination and motor execution a movement-related cortical potential, identified as a negative shift in the electroencephalography (EEG) up to 2 s prior the onset of the movement, can be observed; this is known as the contingent negative variation (CNV). Increases in corticomotor excitability have been demonstrated when the CNV has been used to appropriately pair homologous afferent electrical stimulation (ES) with the onset of movement imagination (Mrachacz-Kersting et al., 2012). This approach has a number of potential benefits over traditional TMS-PAS protocols in that it utilizes the neural circuits active in voluntary movement, can be used in those patients who are routinely contraindicated from TMS based interventions and it has been shown to increase corticomotor excitability with a relatively low number of pairings (Mrachacz-Kersting et al., 2012). However, it is not yet known whether this novel protocol can be used to induce a decrease in corticomotor excitability. Therefore, the current study investigated whether motor evoked potentials (MEPs) in Tibialis Anterior (TA) could be depressed by pairing non-homologous afferent stimulation with movement imagination, and considered the influence of the timing of stimulation relative to movement imagination on the intervention effect.

## Materials and Methods

### Design

An experimental design was utilized, where each subject participated in six experimental sessions held on non-consecutive days. In the first session baseline EEG data during motor imagination was collected to determine the CNV. In sessions 2–4 ES was delivered to the tibial nerve (TN) at three different time points relative to the onset of the imagined dorsiflexion (before, at, after) as determined by the CNV. These session were conducted in a randomized order. Sessions 5 and 6 were control conditions; where session 5 involved ES to the homologous common peroneal nerve (CPN) during imagined dorsiflexion to enable comparison to previously described excitatory protocols (Mrachacz-Kersting et al., 2012) and Session 6 involved ES to the TN without imagined dorsiflexion to investigate the effect of ES alone. All procedures were approved by the New Zealand Health and Disability Northern Y Regional Ethics Committee (ref: NTY/07/05/054) in accordance with the Declaration of Helsinki.

### Subjects

Fourteen healthy subjects without any prior history of sensorimotor dysfunction participated in the study (four females and ten males, 24.6 ± 3.6 years of age). Before participation, all subjects were screened for contraindications to TMS (Rossi et al., 2009) and provided written informed consent.

### Intervention

#### Establishing CNV

In session 1 five channels of monopolar EEG (Grass Model 12 Neurodata Acquisition System Amplifier) were recorded from FCz, C1, Cz, C2, and CPz according to the International 10–20 System using self-adhesive Ag/AgCl electrodes (N Blue Sensor, AMBU A/S). The signals were referenced to the right earlobe and grounded at nasion. The signals were sampled at 1024 Hz and digitized with 32 bits accuracy (CED Power1401 mk 2 Data Acquisition Board). Impedance was accepted if less than 5 kΩ during the recordings. This data was used to establish the CNV from EEG. EEG was analyzed oﬄine using a bandpass filter from 0.1 to 5 Hz with a fourth order Butterworth filter and spatially filtered with a small Laplacian filter with Cz as the center electrode. The peak negativity was determined in each trial in a ±500 ms window around cued task onset and an average peak negativity calculated for each subject.

#### Motor Imagination

**FIGURE 1:**
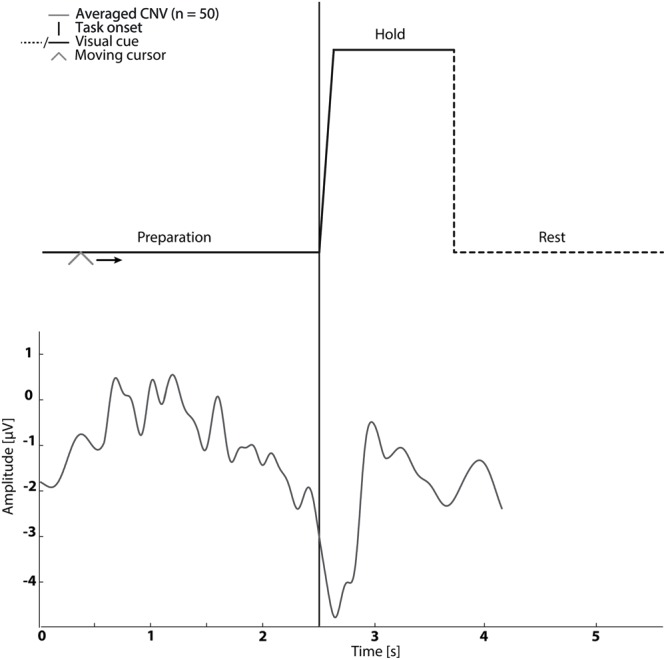
**The visual cue is presented in the top graph.** It was used in Sessions 1–5. An example of the contingent negative variation (CNV) is shown in the bottom graph.

In sessions 1–5 subjects were asked to perform cued kinaesthetic movement imagination of dorsiflexion of the ankle joint. They were cued by a customized Matlab program (see **Figure 1**) which prompted the initiation of imagined movement. The trace in **Figure 1** was shown to the subject while a moving cursor indicated the timing of the different phases. At each session the subject spent approximately 5 min familiarizing themselves with the task before completing 50 repetitions of imagined dorsiflexion.

#### Stimulation Pairing

The mean timing of the peak negativity CNV with respect to the cued task onset was used to determine the timing of paired stimulation. Propagation in the nervous system and cortical processing time was estimated as 50 ms (Mrachacz-Kersting et al., 2007; Kumpulainen et al., 2012). Therefore, in Session 2–4 ES was delivered either; before the onset of motor imagination (mean peak negativity – 50 ms – 2× standard deviation of peak negativity), at the onset of motor imagination (mean peak negativity – 50 ms) and after the onset of motor imagination (mean peak negativity – 50 ms + 2× standard deviation of peak negativity) dependent on the randomization order (see **Figure 1**).

#### Electrical Stimulation

Electrical stimulation (Digitimer Stimulator DS7AH) to the peripheral nerve was delivered at motor threshold (MTh) using a single pulse, with a pulse width of 1 ms via neurostimulation electrodes (Pals Platinum). In TN stimulation (Sessions 2–4 and 6) the cathode was placed distally on the quadriceps muscle, and the anode was placed in the popliteal fossa. In CPN stimulation (Session 5) the cathode was placed inferior to caput fibulae and the anode distal to the cathode to target the deep branch of the CPN. The optimal stimulation site was defined as the site at which ES resulted in activity in the target muscle without any palpable muscle activity in synergistic and antagonistic muscles. Once the optimal stimulation site was determined MTh was determined as the lowest stimulation intensity which elicited a muscle contraction in the target muscle.

### Outcomes

Pre and post-intervention measures of corticomotor excitability were taken in Sessions 2–6 using TMS. Single pulse TMS was used to evoke a MEP in the resting TA using a Magstim 200 figure-of-eight double-cone coil with a posterior–anterior current direction. The MEPs were recorded with Electromyography (EMG) where self-adhesive electrodes (20 mm Blue Sensor Ag-AgCl, AMBU A/S, Denmark) were placed over TA. The EMG was sampled at 4000 Hz and digitized with 32 bits accuracy (CED Power1401 mk 2 Data Acquisition Board). EMG was amplified by custom made amplifiers with a gain of 1000 and bandpass filtered from 20 to 1000 Hz.

The optimal TMS stimulation site was identified by altering the coil position until the site eliciting the largest MEP in the TA muscle was identified (∼2 centimeters anterior to the vertex); the site was marked on the scalp to ensure consistency in coil positioning. The resting threshold (RTh) was determined as the lowest stimulator output that elicited peak-to-peak amplitude greater than 50 μV in five out of ten stimuli. Twelve stimuli were applied at each intensity (90, 100, 110, 120, and 130% of RTh). Each stimulus was separated by 5–7 s. The order of the stimuli was randomized in blocks for each subject.

### Data Analysis

The peak-to-peak amplitudes were extracted for all MEPs and averaged for each TMS intensity. The averaged amplitudes were fitted with the Boltzmann sigmoidal function using the Levenberg–Marquardt non-linear least mean-squares algorithm to obtain a relationship between MEP amplitude and stimulation intensity (Devanne et al., 1997). Four parameters were extracted from the sigmoidal function: (1) maximum peak-to-peak amplitude (MEP_max_), (2) intensity needed to obtain 50% of MEP_max_ (S_50_), (3) slope (K), and (4) *r*^2^-value.

### Statistical Analysis

To establish the stability of pre-intervention measures the averaged MEPs amplitude of pre-intervention measurements obtained in sessions 2–6 were compared using a 1-way repeated measures analysis of variance (ANOVA). To test the effect of timing of TN stimulation on the extracted parameters from the sigmoidal relation between MEP amplitude and stimulation intensity a 2-way repeated measures ANOVA was carried out with factors ‘timing’ (three levels: before motor imagination onset, at motor imagination onset, and after motor imagination onset) and ‘pre/post’ (two levels: pre- and post-intervention). Significant interactions were followed up with one way ANOVAs and/or paired *t*-tests. To test for an effect of CPN stimulation, as well as to test for the effects of TN stimulation alone, separate 2-way repeated measures ANOVAs were carried out with the factors ‘pre/post’ (two levels: pre- and post-intervention) and ‘stimulation intensity’ (five levels: 90, 100, 110, 120, and 130% RTh). Statistical significance was assumed if *P*<0.05, with Bonferroni’s corrections applied to *post hoc* analyses to account for multiple comparisons.

## Results

**FIGURE 2:**
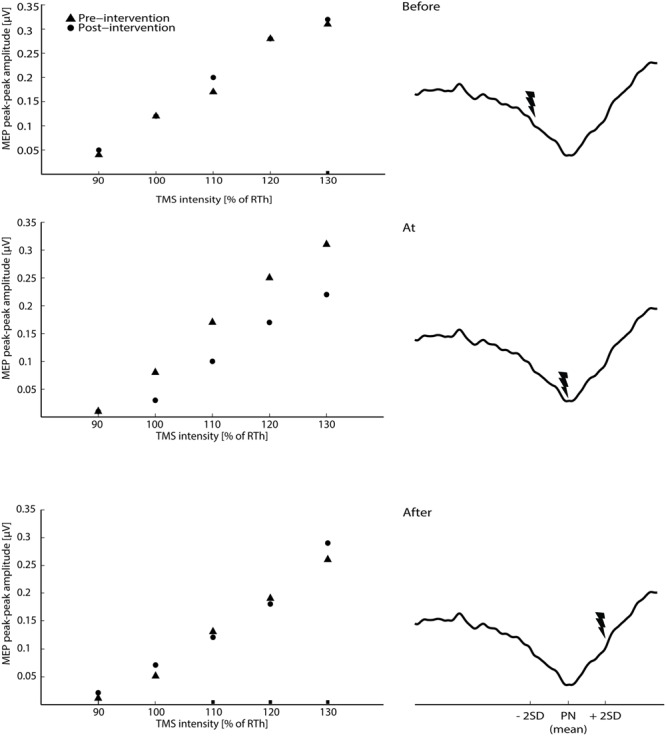
**The raw averaged motor evoked potential (MEPs) for the transcranial magnetic stimulation (TMS) intensities are shown for the three main interventions on the left side of the figure (averaged across all subjects).** On the right site of the figure, the timing of the ES according to the three phases of the CNV (average across all subjects) is shown.

**FIGURE 3:**
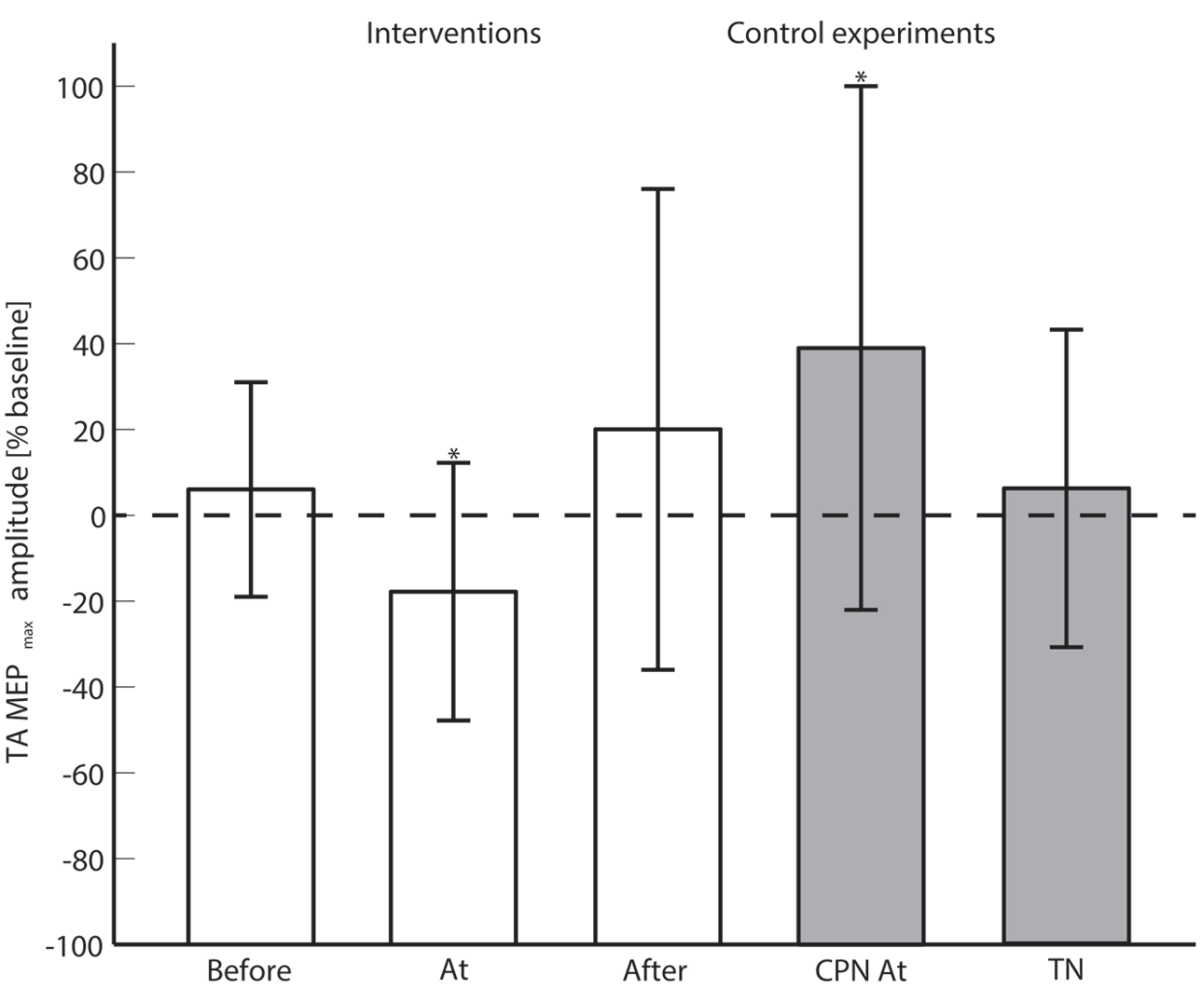
**The averaged (across subjects) MEP_max_ is shown for each of the main interventions (Before, At and and After the onset of motor imagination) and control experiments (CPN stimulation at the onset of motor imagination and TN alone).** 0% change with respect to the baseline means no change from the pre- to post-intervention measurements. ‘^∗^’ indicates a significant difference in the MEP_max_ from the pre- to post-intervention measurements.

The results are summarized in the following three subsections and in **Figures 2** and **3**. The ES of TN was timed according to peak negativity of the CNV recorded in each subject’s baseline session. On average, ES was delivered 705.45 ± 76.93 ms prior to the onset of the task (before motor imagination), 56 ± 289 ms prior to the onset of the task (at motor imagination) and 450.45 ± 103.08 ms after the onset of the task (after motor imagination). There were no significant differences in the size of the MEPs in the pre-intervention measurements across sessions 2–6 [*F*_(8,104)_ = 1.02; *P* = 0.43].

### Effect of Tibial Nerve Stimulation During Motor Imagination

The 2-way ANOVA revealed a significant interaction between the ‘timing’ and ‘pre/post’ [*F*_(2,390)_ = 6.05; *P* = 0.003]. One way repeated measures ANOVA for each timing revealed that there was no significant post-intervention effect for TN stimulation when delivered before [*F*_(1,130)_ = 0.25; *P* = 0.62], or after [*F*_(1,130)_ = 1.15; *P* = 0.23] the imagined movement. However, there was a significant effect when TN stimulation was delivered at the onset of the imagined movement [*F*_(1,130)_ = 11.98; *P* = 0.001], where lower MEPs were obtained in the post-intervention measurements. From the analysis of the extracted parameters from the sigmoidal fit (*r*^2^: 0.82 ± 0.18), a significant decrease of 18% was observed in the MEP_max_ (*P* = 0.039) (see **Figure 3**) and a significant increase in the S_50_ (*P* = 0.022) when TN stimulation was delivered at the onset of imagined movement.

### Effect of Common Peroneal Nerve Stimulation during Motor Imagination

The change in excitability when pairing imagined dorsiflexions with ES of CPN was investigated with a 2-way repeated measures ANOVA with ‘stimulation intensity’ and ‘pre/post’ as factors. The ‘stimulation intensity’ and ‘pre/post’ interaction was significant [*F*_(4,52)_ = 3.11; *P* = 0.023]. *Post hoc* testing revealed a significant effect for ‘pre/post’ [*F*_(1,13)_ = 5.85; *P* = 0.0031] alongside a significant increase in MEP_max_ of 39%.

### Effect of Tibial Nerve Stimulation Alone

To investigate whether the changes in excitability were observed with ES of the TN alone, a 2-way repeated measures ANOVA was performed. The interaction of ‘pre/post’ and ‘stimulation intensity’ was not significant [*F*_(4,52)_ = 0.94; *P* = 0.45]. No significant changes were observed in the parameters of the sigmoidal function.

## Discussion

This study is the first to demonstrate that the excitability of the cortical projections to a target muscle (TA) can be decreased when an afferent stimulation generated through ES of the nerve (TN) innervating the antagonist muscle is paired with motor imagination. The decrease in the excitability (MEP_max_) of TA was 18% of the pre-intervention measurement when stimulation was timed to pair at the onset of motor imagination. This effect is of a similar magnitude to that seen when using conventional TMS-PAS protocols for the upper limb with a short interstimulus interval (10 ms) (Wolters et al., 2003). The difference in the current study from TMS-PAS induced LTD is that cortical activation from motor imagination, an endogenous signal, was paired with ES rather than using an external stimulus such as TMS which activates several circuits through D-wave and I-waves. Importantly in the current study a decrease in corticomotor excitability was achieved with less pairings (*n* = 50) than previous studies which have used traditional PAS protocols (*n* = 90) (Wolters et al., 2003). However, the influence of the number of pairing needs to be investigated further with both traditional PAS and the proposed protocol in this study.

The protocol did not alter corticomotor excitability when afferent stimulation was delivered before or after the onset of motor imagination, indicating that the precise timing of pairing of dissociative stimuli in the cortex and the periphery relative to the onset of motor imagination is crucial. The decrease in corticomotor excitability in the current study was achieved with the same timing relative to motor imagination as that used in homologous agonist–agonist pairing (Mrachacz-Kersting et al., 2012). However, the current study demonstrated a decrease in corticomotor excitability through ES stimulation of the non-homologous muscle. This is in contrast to traditional TMS-PAS protocols where the direction of change is dependent on the interstimulus interval (Stefan et al., 2000; Wolters et al., 2003; Mrachacz-Kersting et al., 2007; Kumpulainen et al., 2012). The effect of motor imagination alone was not tested in this study. However, motor imagination alone has been shown to decrease intracortical inhibition and increase cortical excitability (Pascual-Leone et al., 1995; Abbruzzese et al., 1999). It is possible that in the current study motor imagination obscured the influence of altering interstimulus interval on corticomotor excitability. However, this requires further investigation as previous work has indicated that 50 repetitions of dorsiflexion motor imagery does not induce changes in corticomotor excitability (Mrachacz-Kersting et al., 2012; Niazi et al., 2012).

This study did not seek to determine the mechanism of change in corticomotor excitability, but previous studies investigating ES and PAS protocols using homologous agonist–agonist pairing have suggested that the changes in excitability most likely occur at the cortical level (Wolters et al., 2003; Mrachacz-Kersting et al., 2012; Schabrun et al., 2012). One of the likely areas where the changes may occur is in M1, since afferent feedback may be projected into M1 (Pavlides et al., 1993) at the time when M1 is being activated by motor imagination (Shibasaki and Hallett, 2006; de Vries and Mulder, 2007). Other motor areas are also activated by motor imagination such as the supplementary motor area and the premotor cortex, but the contribution for these areas depends on whether movements are cue-based or self-paced (Lu et al., 2011). However, it is possible that the origin of the changes observed in the current study and mediating mechanisms are different because in the current study non-homologous agonist-antagonist pairing resulted in a pairing of afferent input which was incongruent with the intended motor output.

Little is known about the effect of afferent input which is incongruent with the intended motor output. Studies investigating changes in corticomotor excitability in an antagonist muscle in response to ES alone have demonstrated conflicting results, possibly due to differences in stimulation parameters (Chipchase et al., 2011). The effect of ES alone, without pairing of cortical activation from motor imagination was tested in the current study, but our findings suggest that ES alone is insufficient to modulate the excitability when using such a low number of electrical stimuli (Khaslavskaia et al., 2002; Khaslavskaia and Sinkjaer, 2005). Therefore pairing of non-homologous afferent stimulation with motor imagination seems important in inducing a decrease in corticomotor excitability, presumably overcoming any excitatory effects of motor imagination alone.

Different forms of LTD exist and can be classified as homosynaptic (induced by a conditioned input) and heterosynaptic (induced in a non-conditioned input) (Collingridge et al., 2010). The homosynaptic form of LTD may be consistent with spike-timing dependent plasticity at the system level where temporal asymmetry is important as described for PAS protocols (Ziemann et al., 2004). Besides the temporal asymmetry, another mechanism may be responsible for inducing LTD which is lack of persistence in coincidence as proposed by Stent (1973) as an extension to Donald Hebb’s postulate (Hebb, 1949). This mechanism may be the underlying factor for heterosynaptic LTD that can be observed when, for example, trying to induce LTP in a specific pathway leading to LTD in other inactive pathways (Sjostrom et al., 2008). Homosynaptic and heterosynaptic LTD (or a combination) may be underlying mechanisms for the findings of this study. Several characteristics have been linked to the induction of LTD (Wolters et al., 2003): rapid onset, persistence on cessation of stimulation, reversibility, associativity, *N*-methyl-D-aspartate (NMDA)-receptor dependence and involvement of L-type voltage-gated Ca^2+^ channels. The effects observed in this study fulfill the parameters of induction of LTD in that the onset was rapid, changes were observed after less than 10 min, and they outlasted the stimulation period (the post-measurement was performed approximately 10 min after the cessation of the intervention). It was shown that the changes were reversible since LTP-like plasticity was induced by stimulating CPN instead of TN in a different session (Session 5). The requirement of associativity was also fulfilled as demonstrated by the effect of the timing of ES relative to motor imagination. The last two criteria were confirmed in a PAS study where an NMDA-receptor and L-type voltage-gated Ca^2+^ channel antagonist (Nimodipine and Dextromethorphan) blocked the induction of LTD-like plasticity (Wolters et al., 2003). The NMDA-receptor dependence is important in the model of LTD, which can be depotentiation (Ziemann et al., 2004) or *de novo* LTD (as in the current study); this requires activation at many synapses in the brain (Collingridge et al., 2010).

### Methodological Considerations

The effectiveness of the protocol relied on the assumption that the peak of maximum negativity was stable across trials and over time because the ES was timed according to calculation from data obtained in session 1 for the remaining sessions (2–5). The maximum peak negativity of the CNV has been found to be stable over three recording sessions distributed over three weeks (Mrachacz-Kersting et al., 2012). In that study and the current one, the standard deviation of peak negativity was in the order of a couple 100 ms; this implies that the ES will not be paired with CNV at exactly the same point in time relative to motor imagination in all of the 50 trials. The effect of mistimed afferent volleys is not known, it is possible that greater accuracy in stimulation timing in individual trials may result in either a more potent effect or effect with fewer pairings. It is also not known whether more pairings would result in a greater reduction in corticomotor excitability, however, a prolonged protocol may induce subjects fatigue and subsequent variance in the CNV.

## Conclusion and Implications

This study is the first to demonstrate that the excitability of the cortical projection to a muscle can be inhibited when ES of the nerve supplying the antagonist muscle is paired with imagined movement. Further, this study indicates that the intervention effect is dependent upon the precise pairing of the peripheral afferent stimulation with the onset of motor imagination. Unlike traditional TMS-PAS protocols this intervention is not reliant on TMS and may therefore have utility in a greater number of patients who would normally be excluded from TMS interventions. This protocol has potential in conditions such as spasticity following stroke and focal hand dystonia where neural circuits are over-potentiated due to maladaptive plastic changes. Further refinements, such as the recently developed brain-computer interface which enables online detection of the CNV and pairing of stimulation (Niazi et al., 2012) may further extend the potential of this work.

## Author Contributions

RN collected the data with assistance from IN and MJ. HH, NS, and DT helped interpreting the results. MJ drafted the first version of the manuscript and RN, IN, NS, HH, and DT critically revised the manuscript. All authors approved the final version.

## Conflict of Interest Statement

The authors declare that the research was conducted in the absence of any commercial or financial relationships that could be construed as a potential conflict of interest.
